# Pomegranate action in curbing the incidence of liver injury triggered by Diethylnitrosamine by declining oxidative stress *via* Nrf2 and NFκB regulation

**DOI:** 10.1038/s41598-018-26611-1

**Published:** 2018-06-05

**Authors:** Hadiya Husain, Uzma Latief, Riaz Ahmad

**Affiliations:** Biochemical and Clinical Genetics Lab, Section of Genetics, Department of Zoology, Faculty of Life Science, Aligarh Muslim University, Aligarh, 202002 India

## Abstract

Unearthing and employment of healthy substitutes is now in demand to tackle a number of diseases due to the excessive repercussions of synthetic drugs. In this frame of reference pomegranate juice (PGJ) is a boon comprising of anthocyanins and hydrolysable tannins, known for its anti-oxidant and anti-inflammatory properties. Despite various documented roles of PGJ, there are no studies on antifibrotic potential in NDEA-induced mammalian liver fibrotic model. Hepatic fibrosis in rats was induced by the intra-peritoneal injection of NDEA (10 mlkg^−1^b.wt. of 1% NDEA) in two weeks. Biochemical, histopathological and ultra-structural studies were carried out on control, fibrotic and treated rats. The liver function indices and LPO were increased significantly by intoxication of NDEA. The antioxidant status was disturbed with the decrease in SOD, GST and catalase in the liver and membrane-ATPases as well. Histopathological observations by H&E, M&T, picro-sirius and ultra-structural scrutiny by SEM and TEM indicated liver damage and increase in COX2 and α-SMA by NDEA which was successfully rectified by the supplementation of PGJ. PGJ abrogates liver fibrosis instigated by NDEA in Wistar rats by declining oxidative stress *via* regulation of Nrf2 and NFκB. These findings point towards pomegranate as a potential and efficacious therapeutic agent against liver fibrosis.

## Introduction

The concomitant adverse effects resulting from the employment of synthetic drugs and chemicals have led to the discovery and implementation of efficacious nutraceuticals against diverse diseases. Pomegranate (*Punica granatum*), a native of Western Asia and Mediterranean countries, is a fruit with a worldwide consumption containing compounds beneficial to health^1^. The undesirable skin abnormalities related to aging in humans are abrogated by pomegranate flowers^2^. The flowers and juice of pomegranate have effective hepatoprotective, cardiotonic and anti-inflammatory properties^3–7^ and also inhibits the increased glucose levels in diabetic rats^8^. Seed oil of pomegranate has nephro-protective attributes^9^ while pomegranate peel has been reported to be highly advantageous in being anti-inflammatory and hepato-protective^10,11^. The cancer-chemopreventive property of pomegranate juice and the chemotherapeutic effects against prostate cancer in humans has been reported as well^12^. It has been established by recent studies that pomegranate derived products abrogate chemically induced tumors of lung, skin, breast and colon and it also abates the metastasis of xenografted lung and prostrate tumors in rodents^13^.

Pomegranate has been well documented in relation to the protective role against liver related diseases such as fatty liver in obesity^14^; fatty liver induced by junk food^15^. Assortment of various phytochemicals such as polyphenolic constituents-anthocyanins, hydrolysable tannins-ellagitannins & gallotannins and condensed tannins-proanthocyanidin have been found and reported in diverse parts of the fruit^16^. Pomegranate has exhibited evidences regarding the reduction of oxidative stress mediators which thoroughly directs to its antioxidant ability contributed by its phenolic compounds for its free radical scavenging properties^3,17^. Latterly pomegranate has been much more hyped for its antioxidant activity^18–22^.

Liver fibrosis is a serious health concern which leads to liver diseases such as cirrhosis and primary liver cancer having a worldwide mortality of approximately 1.5 million deaths per year^23^. Now, it is high time that work should be done on these lines to garner the potential of pomegranate in order to cure liver fibrosis. Nitrosamines are a class of chemicals generated during the preservation process by the use of nitrite in foods, latex products and other consumables^24–26^. For this Nitrosodiethylamine (NDEA), a nitrosamine, induced liver fibrosis model in rats is most suitable as some previous studies have reported to generate the disease model in the shortest duration of 14 days^27–29^. Diverse array of hepatocellular injuries including cirrhosis, necrosis, hypertrophy, fibrosis and hepatocellular carcinoma are caused by NDEA^26^. Out of many, liver fibrosis is the main focus of the researchers as the disease may be regressed in its initial stage and the liver can be reversed to normal functioning state. Fibrosis of liver is a dynamic pathological condition which is due to excessive amassing of extracellular matrix emerging from immedicable inflammation. This inflammation ushers the scar tissue formation however it also initiates wound healing process that leads to abatement of inflammatory tissue destruction^30–32^. Main causes of fibrosis in liver are alcoholic steatohepatitis, chronic viral hepatitis B or C infection, autoimmune and biliary diseases^30^. Abstruse etiological causes of liver diseases remains as obscure, however the elementary component in pathogenesis of liver diseases is oxidative stress^33^. Various lines of researches have recently substantiated and evinced the crucial role of oxidative stress in the pathogenesis of liver fibrosis^34–36^ and hence; the prevention of liver fibrosis is possible by the efficient antioxidants^37–39^. Use of chemically induced and clinically relevant liver fibrosis model for elucidation of pomegranate potential against liver fibrosis is highly significant because there is a general paucity of documentation on pomegranate juice against liver fibrosis. However a few reports on pomegranate against NDEA-induced hepatocarcinogenesis^40^ and hepatotoxicity^41^, and CCl4- induced oxidative stress^42^ has been presented. Pomegranate peel and seed extracts have been reported to have protective effects against CCl_4_-induced liver fibrosis^43^, the ameliorative effect of pomegranate juice (PGJ) against liver fibrosis induced by NDEA has not yet been documented till date.

Exploration of ameliorative mechanism by PGJ using ultra-structural characteristics, assessment of antioxidant enzymes, analysis of lipid peroxidation and membrane ATPases, western blotting of Nrf2, NFкB along with evidentiary support of histopathology, immunohistochemistry of Cycloxygenase-2 (COX2), Alpha-smooth muscle actin (α-SMA) and electron microscopy pragmatically suffice our novel approach. It is imperative to mention that the present study has a much greater relevance to other studies related to pomegranate against liver fibrosis as juice of arils has been used which is widely consumed in most of the countries worldwide. Therefore, the present study is the first to elucidate the possible role of PGJ in ameliorating NDEA-induced hepatic fibrosis in Wistar rats. Further, this study also explores a plausible mechanism of action to establish PGJ as a potential therapeutic agent.

## Materials and Methods

### Chemicals and reagents

Acrylamide, adenosine 5′ Triphosphate, 3,3′-diaminobenzidine, Bis-acrylamide, ammonium per sulfate (APS), TEMED, N′-Nitrosodiethylamine (NDEA), picrosirius red, tris-buffer, CBBR-250, perchloric acid, thiobarbituric acid (TBA), trichloroacetic acid (TCA), Sodium dodecyl sulfate (SDS) were procured from Sigma-Aldrich chemicals Pvt. Ltd. (St. Loius MO). Pyrogallol, hematoxylin and eosin, bovine serum albumin (BSA) were obtained from SRL (Mumbai, Maharashtra, India). LFT assay kits were purchased from AutoZyme (Accurex Biomedical Pvt. Ltd, Mumbai, India), Erba Diagnostics Mannheim Gmbh (Mumbai, India) while all other reagents and chemicals were of analytical grade. NFкB p65 and Nrf2 Rabbit polyclonal antibodies were from Santa Cruz Biotechnology, while Goat anti-Rabbit IgG antibody, (H + L) HRP Conjugate polyclonal secondary antibodies were from Sigma. Immobilon western chemiluminescent HRP substrate was from Merck Millipore. Polyclonal α-SMA antibodies were obtained from Trends Bio-product Pvt. India and COX2 polyclonal mouse antibodies were purchased from Cayman chemicals.

### Pomegranate juice preparation

Two kilograms of pomegranates (*Punica granatum* var. Bhagwa) were brought to the laboratory and identified for its taxonomic position by the experts in the Department of Botany of this University. The fruits were manually washed; peeled and separated arils were processed to extract the juice. Using a commercial blender, 150 ml of red colored juice was obtained that was allowed to filter through Buchner funnel for 7–8 hrs at 4 °C under hygienic conditions. Filtration minimized the yield up to 10–15%. The pomegranate juice (PGJ) was used afresh or stored at −20 °C for further investigations for a maximum period of two weeks^44^.

### Animals

Adult male Wistar rats (*Rattus norvegicus*) weighing 140 ± 10 gm (6–7 weeks) were the subject of this study. Animals were kept in polycarbonate cages with a wire mesh top in a room under standard conditions of illumination and at 25 ± 2 °C. They were provided with proper sterilized diet and water *ad libitum*. The animals were kept in clean and hygienic conditions. The study synopsis was scrutinized by the Departmental board of studies that was finally approved by the Committee for the Advance Studies and Research (CASR). The experiments were in accordance with the guidelines of the Committee for the Purpose of Control and Supervision of Experiments on Animals (CPCSEA), India.

### Experimental protocol and treatment schedule

The animals used in this study were divided into seven experimental groups with five healthy rats each. Group-I, was given normal saline for a period of 14 days; Group-II & -III, were supplied with fresh PGJ (i.p.) in 2 ml/kg b.wt^45^ thrice a week for 7 and 14 days and sacrificed on the 7^th^ and 14^th^ day respectively; Group-IV & -V, were administered (i.p.) NDEA with a single dose of 10 ml/kg b.wt of 1% NDEA solution injected (i.p) and were sacrificed on the 7^th^ and 14^th^ day respectively^29^. Groups-VI & -VII were given a single dose of NDEA followed by repeated administration of PGJ thrice a week in the doses described above and sacrificed on 7^th^ and 14^th^ day respectively. Approximately 2 hrs lag was maintained in NDEA and PGJ treatment based on some pilot experiments and other reports.

### *In vitro* experiments on antioxidant activity of pomegranate

#### 2,2-diphenyl-1-picrylhydrazyl radical (DPPH) assay

The stable DPPH was used in determining the free radical scavenging property of PGJ^46,47^. 100 µl of a 0.2 mM DPPH stock solution was added to 100 µl each of standard 5 mM ascorbic acid (control) and PGJ in separate tubes and mixed for 5 sec. Each reaction mixture was kept in dark at 18 °C for 30 min to reach steady state. The absorbance values were recorded spectrophotometrically at 515 nm in a cuvette. The results were expressed as the percentage inhibition of the DPPH.

$${\rm{Antioxidant}}\,{\rm{capacity}} \% =[({\rm{Absorbance}}\,{\rm{Control}}-{\rm{Absorbance}}\,{\rm{Sample}})/{\rm{Absorbance}}\,{\rm{Control}}]\times 100$$

#### Determination of phenolic content

The total phenolic content in the fresh PGJ was determined according to the method of Folin-Ciocalteau in which the blue colored reaction product was used to determine the phenolic content^48^. Briefly, this method includes the reduction of phosphorwolframate-phosphomolybdate complex. The total volume of the reaction was miniaturized to 1 ml. 20 µl of diluted PGJ with 1580 μl water and 100 µl of Folin–Ciocalteau reagent were mixed thoroughly. Subsequently, 300 µl of 20% Sodium carbonate (Na_2_CO_3_) was added. The reaction mixture was incubated for 2 hrs at room temperature in dark. Absorbance was recorded at 765 nm. Gallic acid was used as standard to extrapolate unknown values and expressed as gallic acid equivalents in mg per liter of PGJ (mg GAE/L of PGJ).

#### Ferric reducing antioxidant power (FRAP) assay for pomegranate juice

The total antioxidant activity of PGJ was measured using the protocol of Benzie and Strain^49^. FRAP assay uses antioxidants as reductants in a redox-linked colorimetric method, employing an easily reduced oxidant system present in stoichiometric excess. Briefly, FRAP reagent including 10 mM 2,4,6 Tripyridyl-S-triazine (TPTZ), 20 mM FeCl_3_ and 300 mM acetate buffer of pH 3.6 was prepared. FRAP reagent was incubated at 37 °C for 10 min. Ascorbic acid calibration curve was drawn by taking different known concentrations of it. 33.33 µl of standard ascorbic acid and PGJ sample was added to 966.7 µl of FRAP reagent separately. Both the reaction mixtures were mixed well and their optical densities were recorded separately at 593 nm. FRAP values were expressed as µM equivalents of ascorbic acid.

### Sera collection and tissue sampling

Rats were anaesthetized in chloroform and blood was collected by cardiac puncture before their sacrifice on the 7^th^ and 14^th^ day. For the extraction of sera our own established protocol was followed^50^. Serum samples were analyzed afresh for the assessment of liver function test. Sterilized scissors and forceps were used to excise liver from the sacrificed rats. Phosphate buffer saline (50 mM, pH 7.0) was used to remove tissue debris from the excised liver and blotted individually on ash-free filter paper. The liver tissues were homogenized in 1:3 w/v Tris-HCl buffer (50 mM, pH 7.5), unless otherwise mentioned specifically. To obtain clear supernatants, the crude homogenates were centrifuged at 8000 rpm at 4 °C for 15 min and stored in aliquots at −20 °C until further analyses.

### Liver transaminases (AST, ALT), ALP, γGT and bilirubin assay

Freshly collected sera were processed to examine aspartate transaminase (AST), alanine aminotransferase (ALT), alkaline phosphatases (ALP), gamma glutamyltransferase (γGT), and bilirubin levels. The estimation of enzyme concentrations was strictly accorded the procedures as described in the instruction manual of Erba Diagnostics Mannheim Gmbh (Mumbai, India) and AutoZyme (Accurex Biomedical Pvt. Ltd, Mumbai, India).

### Protein estimation

The total protein content was estimated according to the protocol described by Lowry^51^. Folin-ciocalteau’s phenol was used as color reagent and the unknown protein concentrations were extrapolated against the known molarities of bovine serum albumin (BSA). Absorbance (optical density) was read at 660 nm on a UV-visible spectrophotometer.

### Densitometry and quantitative assessment of sera albumin

Alterations in sera albumin were observed quantitatively on 10% acrylamide gels in the presence of SDS. Bands were visualized after overnight washing of gels in 5% acetic acid and staining by Coomassie Brilliant Blue (CBB-R250). Protein ladder in the range of 14.3 to 203 kDa was used as standard. Stained PA gels were further assessed by densitometry analysis using the GelPro (Media Cybernetics, USA) and Scion Imaging software (Scion Corporation; Beta release, 4.0) programs.

### Lipid peroxidation assay

Lipid peroxidation (LPO) in liver tissue was estimated in terms of malondialdehyde (MDA) by following the method of Ohkawa^52^. To a total of 3.4 ml reaction mixture consisted of 0.2 ml of 8.1% sodium dodecyl sulfate (SDS), 1.5 ml of 20% acetic acid (pH3.5) and 1.5 ml of 0.8% aqueous solution of thiobarbituric acid (TBA), 0.2 ml of crude liver homogenate and raised to 4 ml with water. Following heating for 1 hr at 95 °C, the mixture takes a pink color that was cooled until it attained room temperature. Then, 5 ml of n-butanol and pyridine mixture (15:1 v/v) was added to the solution and vortexed. The contents were centrifuged at 8000 rpm for 10 min and the absorbance of organic layer was read at 532 nm. The extent of lipid peroxidation was measured in terms of the concentration of MDA and expressed as nmol/g of liver tissue using an extinction coefficient of 1.56 × 10^5^ M^−1^ cm^−1^.

### Superoxide dismutase estimation

Superoxide dismutase (SOD) activity was determined by the established protocol of Marklund and Marklund^53^ and the value was calculated by following the method of Nandi and Chatterjee^54^. The activity was measured by preparing a reaction mixture consisting of 2.75 ml of 50 mM PO_4_ buffer (pH 8.5), 0.1 ml of 1 mM EDTA and 50 µl of liver tissue homogenate which was incubated for 20 min at 25 °C. 0.1 ml of 0.2 M pyrogallol was added to the mixture under dark and the difference in the optical densities were recorded after every 60 sec for 3 mins at 420 nm. The specific activity of the reduction of pyrogallol auto-oxidation was determined as Units/mg protein/min.

### Determination of Catalase activity

The ability of catalase to breakdown H_2_O_2_ was employed in the measurement of its activity following the method of Aebi^55^. Briefly, the reaction mixture consisted of 1.9 ml of 50 mM phosphate buffer (pH 7.0), 0.1 ml of liver supernatant and 1.0 ml of freshly prepared 30 mM H_2_O_2_. The decomposition rate of H_2_O_2_ was recorded immediately at 240 nm after an interval of 60 sec for 3 min.

### Estimation of Glutathione-S-transferase activity

Determination of Glutathione-S-transferase (GST) activity was carried out in accordance with the protocol of Habig^56^. The reaction mixture contained 500 µl of 0.1 M phosphate buffer (pH 6.0), 200 µl of 10 Mm reduced glutathione, 150 µl of 10 mM of 1-chloro-2, 6-dinitrobenzene (CDNB) and 50 µl crude homogenate of liver tissue. The Δ absorbance was recorded at 340 nm for 3 min and expressed as µmol GSH-CDNB conjugate/min.

### Determination of Na^+^/K^+^-ATPase, Mg^2+^ ATPase and Ca^2+^ATPase activities

Membrane-bound ATPase activities were determined by following the established protocol of Ahmad and Hasnain^57^. Color was developed by the addition of 0.5 ml of ammonium molybdate and 0.25 ml of 1 -Amino-2-naphthol 4-sulfonic acid (ANSA) and incubated at 25 °C for 20 min. Absorbance of the purple to dark blue colored mixture was read at 640 nm.

### Assessment of Glucose-6-phosphate dehydrogenase activity (G6PD)

Activity of G6PD was determined spectrophotometrically^58^. Reagents including NADP (1.08 × 10^−4^ M), NBT (6 × 10^−4^ M), PMS (17 × 10^−3^ mM) and Tris–HCl (125 mM) were mixed with 25 μl of liver homogenate of each group. The reaction was initiated by finally adding up G_6_PD Na_2_G_6_.PO_4_.H_2_O (16 × 10^−4^ M) making the reaction mixture volume up to 2 ml each. The optical densities were recorded at 540 nm after 40–50 min incubation period at room temperature.

### Detection of Nrf2 and NFκB by Western Immunoblotting

Initial screening was done by Polyacrylamide gel electrophoresis and vertical slabs electrophoretic runs (10 × 10 × 0.1) cm^3^ were made in 10% acrylamide according to the protocol of Laemmli^59^. Expression levels of Nrf2 and NFκB were discerned by Western immuno-blotting using the protocol of Sambrook *et al*.^60^. Equilibration of SDS-PA gels was done for 25–30 min in transfer buffer (194 mM glycine, 24 mM Tris and 10% v/v methanol). PVDF membranes (0.45 mm, BioRad, USA) were used for electrotransfer at 125 V/200 mA for 2 h at 4 °C. Membranes were washed three times in PBS (50 mM, pH 7.1) and treated with blotto (5% w/v non-fat dry milk in PBS) for about 1 h at room temperature. The blocking solution (i.e blotto) consisting of 0.1% Tween-20 (PBS-T) (w/v) was used for the remaining steps of incubation and washing. Incubation of the blots with gentle shaking for 2 hrs was done separately in primary antibody (Nrf2 antibody and NFκB antibody in dilution 1:500 and 1:400 respectively) and washed three times in PBS-T for 30 mins each. Secondary antibody (HRP-conjugated Goat anti-Rabbit IgG, dilution of 1:200) was used for treatment of membranes for almost 2 hrs at room temperature. For removal of unbound antibody the membrane was washed in PBS-T thrice. Immobilon western chemiluminescent HRP substrate was used for detection of signals. Densitometric analysis of the blot scans was done using the GelPro (Media Cybernetics, USA) and Scion Imaging (Scion Corporation, Beta release, 4.0) software programs.

### Histopathology

Liver specimens were collected from the tissue and kept in 10% formalin solution. After fixation the tissues were processed by serial dehydration, clearing in xylene and then embedded in paraffin wax. 5 µm slices were sectioned and stained by routine Hematoxylin and eosin (H&E) and Masson’s trichrome (M&T). Picro-sirius red was used to stain the liver tissue sections to visualize the collagen content under fluorescence at 475–500 nm^61–63^. Photomicrographs were taken on Zeiss Axioscope A1 with Jenoptik Prog Res C5 camera model and Zeiss Axioscope 40.

### Scanning electron microscopy

The liver tissue sections were precisely cut to the size of 1 mm^3^ and fixed for overnight in 2.5% gluteraldehyde in 0.1 M sodium phosphate buffer (pH 7.4). 1% Osmium tetraoxide was used for staining for 1 hr. These tissue sections were further processed by a stepwise dehydration with ethanol gradient at a gap of 10 mins and vacuum dried for overnight^64^. The stubs were applied with sputtered metal coatings of gold and the observations were captured using the field emission scanning electron microscope (FE-SEM, JEOL JSM6510LV) at 10 kV.

### Transmission electron microscopy

Fresh liver samples were processed for transmission electron microscopy (TEM) by fixing in 2.5% gluteraldehyde followed by washing in phosphate buffer saline (pH 7.4) three times to be finally fixed in 1% Osmium tetraoxide for 1 hr at 4 °C. Further processing included established dehydration in graded series of ethanol, embedding in epoxy resin and then cutting into ultrathin sections. Mounting of sections on copper grids was done followed by staining with uranyl acetate and lead citrate and observed under a transmission electron microscope (JEOL JEM-2100) at 100 kV^65^.

### Immunohistochemistry of COX2 and α-SMA

For immunohistochemistry of COX2 and α-SMA, serial liver sections were processed according to the protocol of Ahmad *et al*.^66^. Briefly, endogenous peroxidase activity was quenched by 3% H_2_O_2_ prepared in methanol followed washing in PBS (0.01 M PBS, pH = 7.2). The slides were then incubated in BSA (3%) and then washed again in PBS. Then, the slides were incubated with primary antibody i.e. COX-2 polyclonal mouse antibodies (1:200) and α-SMA polyclonal mouse antibodies (1:400) for overnight at 2–8 °C in a moist chamber. Unbound antibodies were washed off with PBS before incubating with secondary antibody (HRP-conjugated goat antimouse IgG immunoglobulins; 1:500) at RT for 50–60 min. After washing in PBS, the sections were developed using freshly prepared 3% 3,3′-diaminobenzidine tetrahydrochloride hydrate (DAB) solution. The stained sections were rinsed with PBS, counterstained with Harris hematoxylin and mounted with DPX (distyrene, plasticizer and xylene mixture). Stained slides were then photographed under Zeiss Axioskop 40.

### Statistical analysis

All the biochemical comparisons between groups were conducted by student’s t-test using SPSS. A level of p ≤ 0.05 was considered statistically significant. Baseline characteristics are presented as mean ± standard deviation for the continuous variables.

### Data availability statement

All data generated and analyzed during this study are included in this published article.

## Results

### *In vitro* studies on the antioxidant activity of pomegranate

Freshly prepared pomegranate juice (PGJ) was processed to determine its antioxidant ability *in vitro*. This study discernibly indicated that PGJ contains total phenolics of the magnitude of 1634 ± 24 mg of gallic acid equivalents/L. The free radical scavenging property of PGJ was measured as the percentage antioxidant capacity which came out to be 55.9%. Further, FRAP assay revealed the antioxidant capacity of the pomegranate juice that was found to be ~1202 µM equivalents of ascorbic acid.

### Effect of Pomegranate juice on investigated biochemical parameters

**Figure 1:**
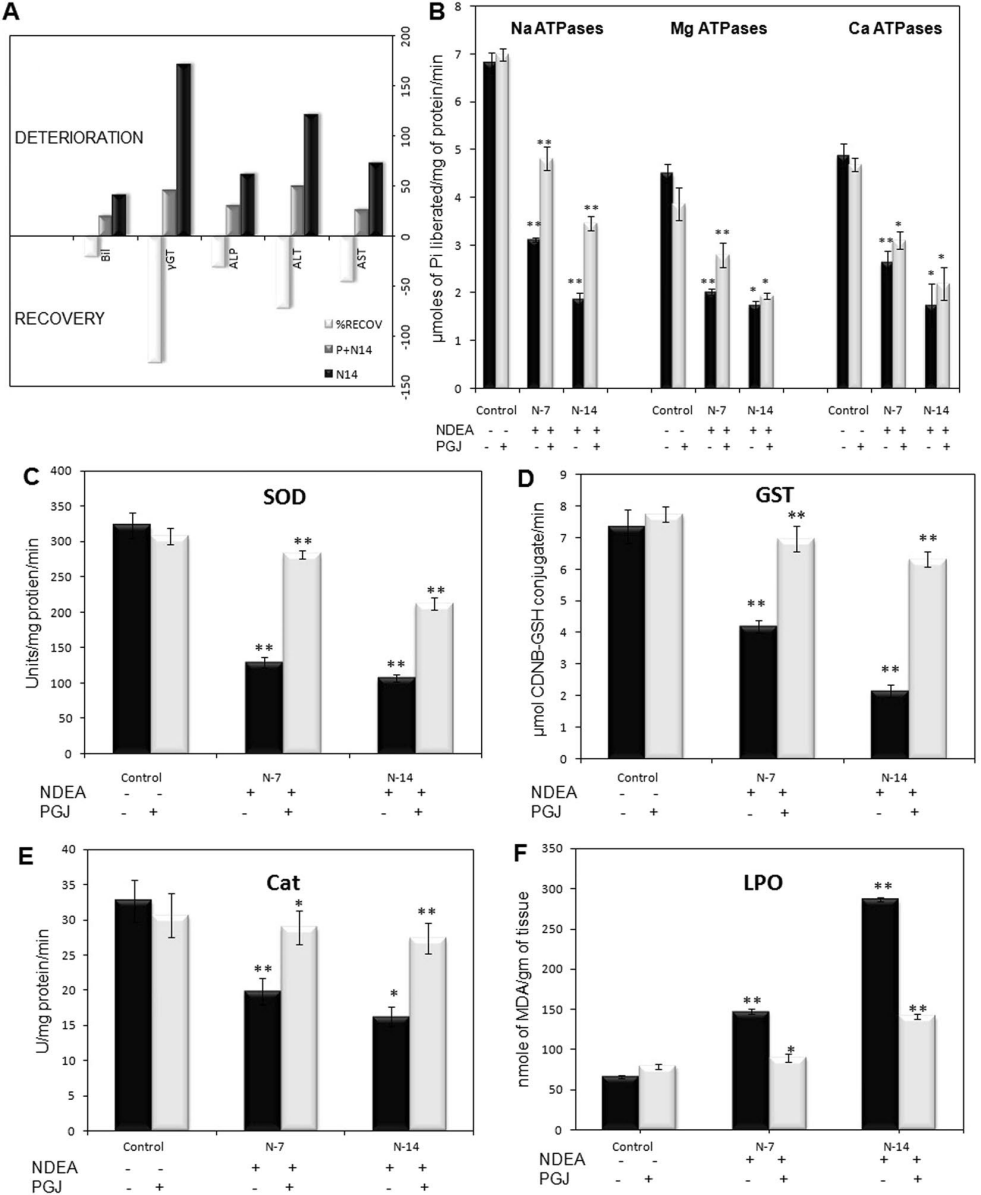
**(A)** Bar graph showing relative change in the liver function parameters as deterioration phase (NDEA treated) and recovery phase (PGJ supplemented) in male rats. AST = Aspartate aminotransferase (IU/L), ALT = Alanine transaminase (IU/L), ALP = Alkaline phosphatase (IU/L), γGT = Gamma-glutamyl transpeptidase (IU/L), B = Bilirubin (mg/dL). The values are mean of five assays. **(B)** ATPases activity in liver. Activities of membrane-bound ATPases in the liver of rats treated with NDEA and NDEA + PGJ, along with their respective controls (Saline/PGJ) during the progression of the treatments. Each bar represent the mean ± SD value (n = 5) of experiments performed in triplicates (*P < 0.05; **P < 0.01). Bar diagram showing the hepatic levels of **(C)**. Superoxide dismutase (SOD, U/mg of protein/min) **(D)**. Glutathione-S-Transferase (GST, µmol CDNB-GSH conjugate/min) **(E)** Catalase (U/mg protein/min) and **(F)**. Malondialdehyde (MDA, nmoles/g tissue) of rats treated with NDEA and NDEA + PGJ, along with their respective controls (Saline/PGJ) during the progression of the treatments. Each bar represent the mean ± SD value (n = 5) of experiments performed in triplicates (*P < 0.05; **P < 0.01).

In this study, non-significant differences were observed in general among the saline control and PGJ supplemented groups. Though insignificant increase was observed in few of the investigated parameters between the PGJ supplemented day-7 and day-14 specimens. Thus, in the subsequent description we have used the term ‘control’ only to compare the data with the NDEA-treated animals. The levels of the liver function indices *i*.*e*. enzymes of hepatic damage ALT, AST, γGT, ALP and bilirubin increased significantly in day-14 NDEA treated rats in comparison to the control (P < 0.05). Further, these parameters differ significantly between NDEA treated day-7 and day-14 samples. The treatment of PGJ abrogated the liver injury by decreasing these enzyme levels in rats in a time and dose-dependent manner (Fig. 1A).

Perpetual increase in hepatic tissue damage was further validated by the determination of membrane-bound ATPases. Our study revealed a significant amount of decline in Na^+^/K^+^-ATPase, Mg^2+^-ATPase and Ca^2+^-ATPase due to NDEA-induced intoxication in rats within two weeks as compare to control. Supplementation of PGJ restitutes the levels of these ATPases towards normal in a dose and time-dependent manner during the course of treatment (Fig. 1B).

The hepatic antioxidant enzyme activities of SOD, GST and catalase were found to be depleted in the experimental animals belonging to the NDEA-treated groups. Rectification of SOD, GST and catalase enzyme levels by the application of PGJ treatment in rats was clearly evident by the significant increase in their levels within two weeks of treatment. Lipid peroxidation in the membranes was estimated by variations in malondialdehyde levels. In time-dependent manner, NDEA treatment elevated the levels of MDA significantly in two weeks compared with controls (Fig. 1C–F). Administration of PGJ alleviates the peroxide formation in the animals and facilitates improved hepatic functioning, as also revealed by LFT biomarkers.

### Changes in serum albumin levels

**Figure 2:**
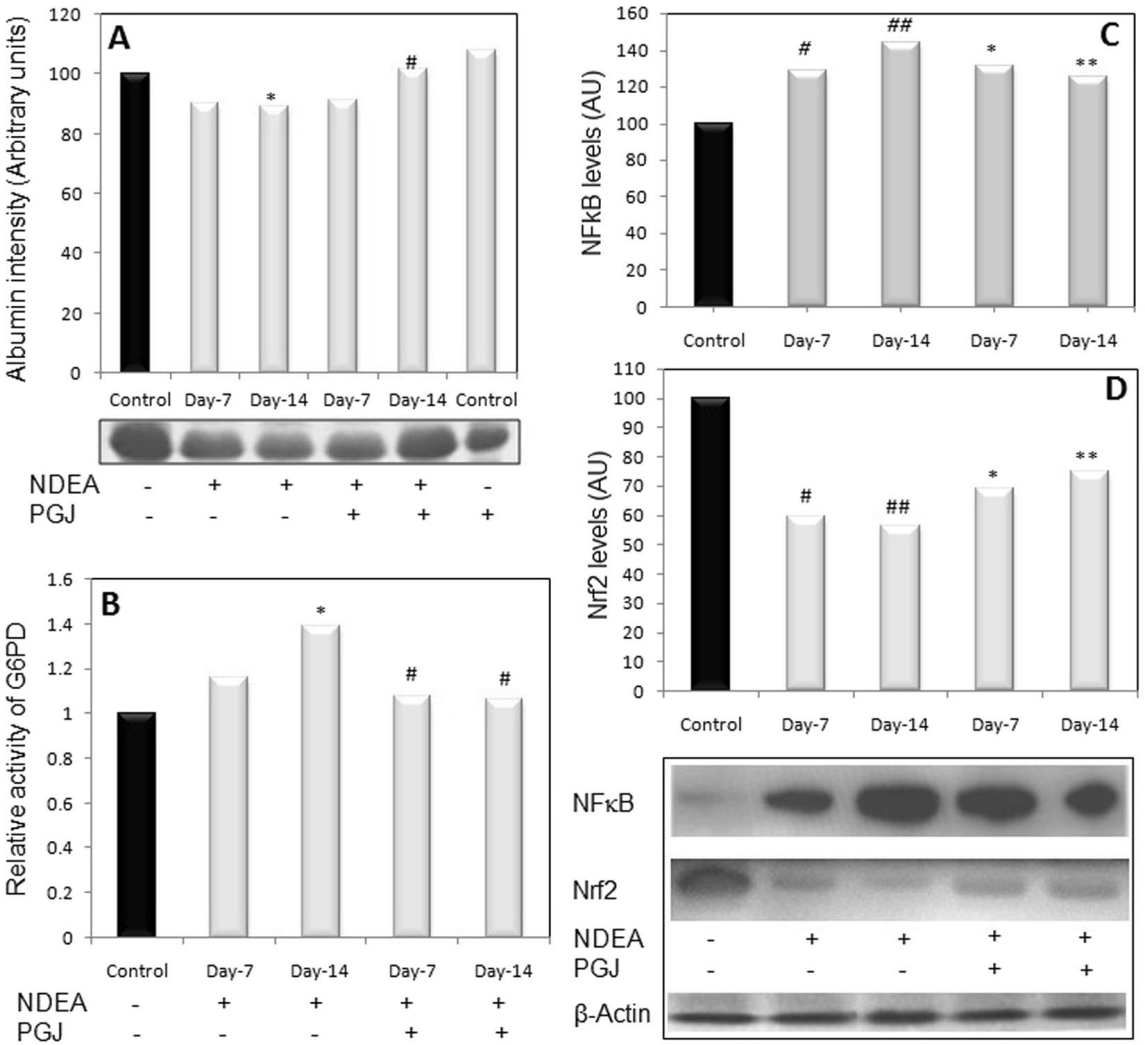
**(A)** Image showing changes in the levels of sera albumin in control and NDEA treated samples of rats. Bar graph shows quantitative variations in the expression of albumin protein (^*^P < 0.05 versus control; ^#^P < 0.05 versus NDEA Day−14 treated *i*.*e*. Fibrotic). **(B)** The bar diagram illustrates quantitative differences in the levels of G6PD between control and treated groups of animals (^*^P < 0.05 versus control; ^#^P < 0.05 versus NDEA Day-14 treated *i*.*e*. Fibrotic). **(C)** Western immunoblot image showing NFкB expression in the liver of NDEA treated (Day-7 and 14), PGJ supplemented and control group animals. ^#^P < 0.05 and ^##^P < 0.01 versus control; *P < 0.05 and **P < 0.01 versus NDEA Day-14 treated *i*.*e*. Fibrotic). **(D)** Western immunoblot image showing Nrf-2 expression in the liver of NDEA treated (Day-7 and 14), PGJ supplemented and animals belonging to control groups. ^#^P < 0.05 and ^##^P < 0.01 versus control; *P < 0.05 and **P < 0.01 versus NDEA Day-14 treated *i*.*e*. Fibrotic).

Significant fluctuations were observed in the serum albumin levels among different groups. Prominent decrease in the levels of sera albumin of NDEA supplemented rats was evident in comparison to the control group. In contrast the levels were notably restored after pomegranate juice supplementation as compared to day-14 fibrotic animals. (Fig. 2A).

### Adjustments in G6PD levels

Activity of G6PD was found to be significantly increased at day-14 by ~40% in NDEA intoxicated fibrotic rat groups with respect to the control. Further there was a dramatic decline by ~24% in day-14 in the livers of pomegranate supplemented animals in comparison to day-14 NDEA group, clearly indicating prominent abatement of hepatic fibrosis. (Fig. 2B).

### Changes in the hepatic Nrf2 and NFκB levels

The western immunoblot profiles of the tissue homogenates exhibited significant quantitative differences among different treatment groups. Western-blot analysis reveals significant decrease in the Nrf2 expression at day-14 by ~44% in NDEA-induced fibrotic animals compared with control. PGJ supplement restores Nrf2 levels in NDEA-treated rats by ~19% within two weeks in comparison to day-14 NDEA treated fibrotic rats. Conversely, a significant increase was found in the levels of NFκB in NDEA-induced fibrotic rats at day-14 by ~42% compared with control. PGJ supplement restores NF-кB levels within two weeks by ~20% in comparison to day-14 NDEA treated fibrotic rats (Fig. 2C,D).

### Histopathological and Ultra-structural changes in the liver

**Figure 3:**
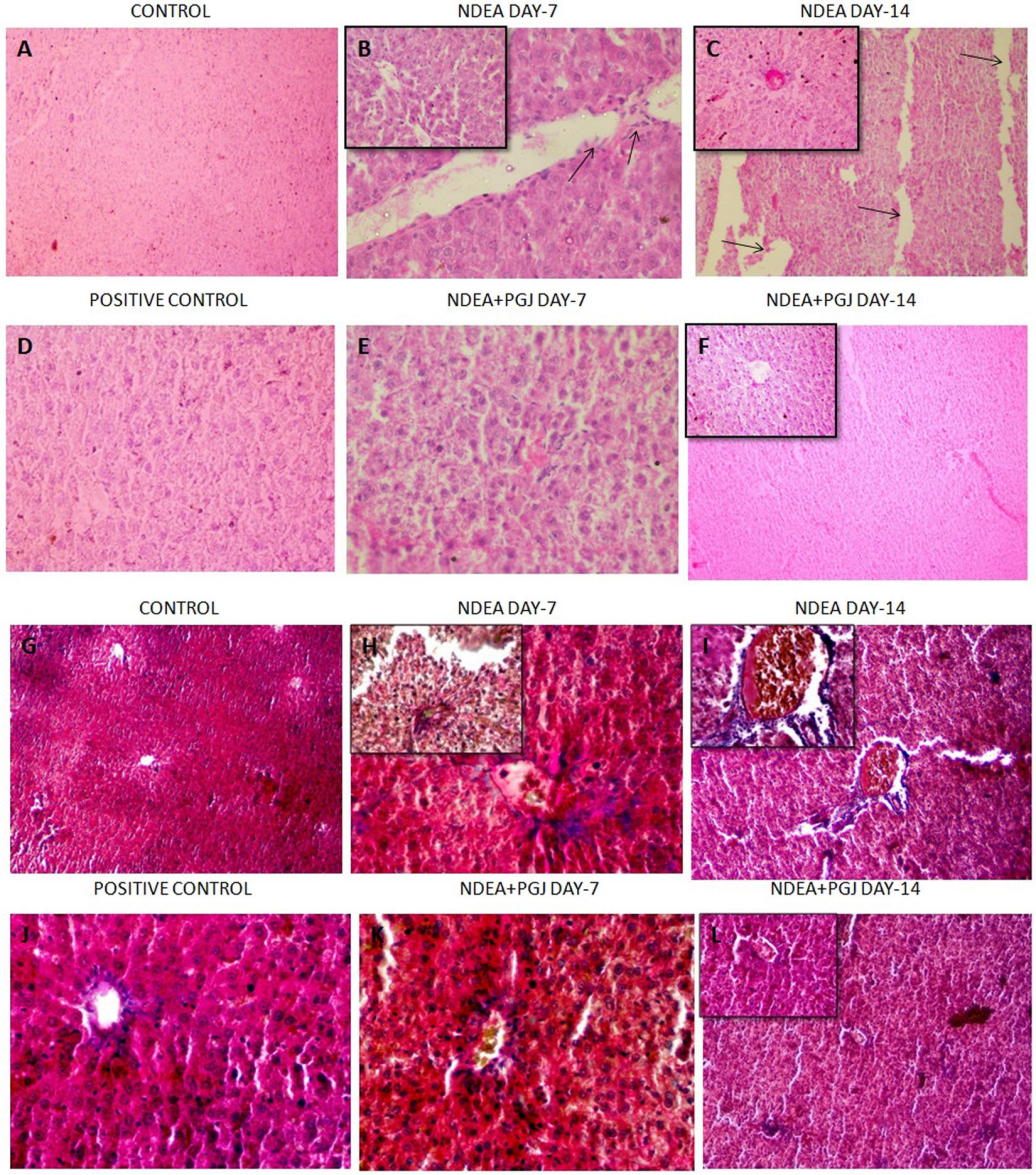
H&E staining of rat liver. **(A)** Normal liver architecture observed in animals of control group (10X). **(B)** NDEA Day-7 showing hepatic necrosis, centrilobular congestion and congestion of sinusoids with neutrophilic infiltration indicated with arrows (40X). (Inset)- Multiple subsidense of the liver parenchyma (40X); **(C)** NDEA Day-14, Colossal dilation of sinusoids and hemorrhagic necrosis of the central zonal hepatocytes. Fibrous expansion of portal tracts (10X). (Inset)- Increased arteries and microvessels in centrizonal scars (40X). **(D)** PGJ control rat liver section exhibit normal liver architecture with thinner sinusoidal spaces and absence of portal mononuclear infiltrates (40X). **(E)** PGJ + NDEA Day-7 sections exhibit lesser sinusoidal spaces (40X). **(F)** PGJ + NDEA Day-14 rat liver sections profoundly exhibit recovered cellular organization of liver and lobular framework (10X). (Inset)- Intact centrilobular area adjacent to central vein (40X). M&T staining of rat liver. **(G)** Normal liver architecture and absence of any blue stained collagen fibers in rats of control group (10X). **(H)** NDEA Day-7, blue colored collagen fibers observed around the central vein exhibiting expanding portal and periportal fibrosis (40X) Inset-(40X). **(I)** NDEA Day-14, liver inflammation in central vein and abundance of blue colored collagen fibers around the central vein and centrilobular necrosis with new vascular connections between portal fields and central veins (20X). **(J)** PGJ supplemeneted control rat liver section exhibit normal liver architecture with absence of blue colored collagen fibers (40X). **(K)** Refurbishment of liver architecture in the rat liver sections supplemented with both NDEA and PGJ in 7 days (40X). **(L)** PGJ + NDEA Day-14 liver specimens show evident attenuation by PGJ noticed by the absence of blue stained collagen fibers (10X).

**Figure 4:**
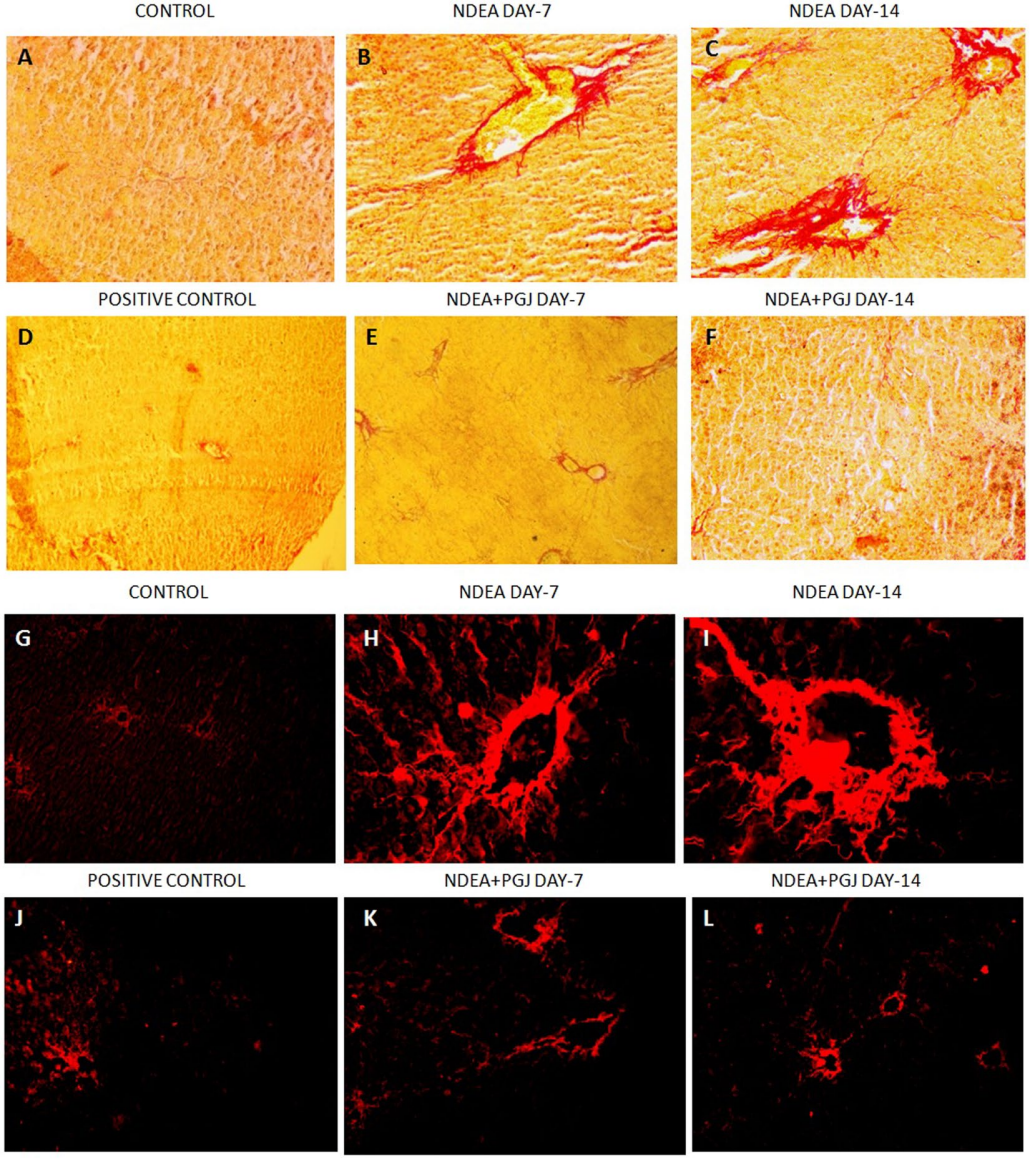
Rat liver sections stained with picrosirius red observed under bright field microscopy. **(A)** Rat liver sections exhibit preserved architecture with normal hepatocytes (20X). **(B)** NDEA Day-7, bright red colored collagen fibers present in the sinusoidal region (20X). **(C)** NDEA Day-14, bridging fibrosis observed by the red colored collagen fibers extending from new vascular connections between portal fields and central veins (20X). **(D)** PGJ supplemented control rat liver section showing unaltered liver architecture (10X). **(E)** Abatement of liver architecture in rats treated with NDEA along with PGJ for 7 days (20X). **(F)** Rat liver specimens supplemented by both NDEA and PGJ for 14 days show restored liver structure (20X). Rat liver sections stained with picrosirius red observed under fluorescent microscopy. **(G)** Saline control liver sections showed traces of red colored collagen under fluorescent microscopy (10X). **(H)** NDEA Day-7, demonstrating collagen accumulation (red colored) (40X). **(I)** NDEA Day-14 specimens exhibiting relatively more dense collagen fibers impregnated into the sinusoidal spaces (40X); **(J)** Positive control (PGJ treated) rat liver sections show trivial amount of red colored collagen (10X). **(K)** NDEA + PGJ Day-7, liver sections exhibit profound decline in the collagen content as compared to the NDEA intoxicated animals (10X). **(L)** NDEA + PGJ Day-14 liver specimens showing significant reduction in the amount of collagen contents a visible under fluorescence microscopy (10X).

Normal lobular architecture with normal hepatocytes, central veins and radiating hepatic cords in control group of rats were exhibited by H & E and Masson’s trichrome staining (Fig. 3A,G). NDEA treated liver sections of rats sacrificed on day-7 displayed slight histological changes such as dilated central veins, hemorrhage and diffuse centrilobular congestion (Fig. 3B,H). Day-14 liver sections of NDEA treatment evinced a more profound tissue damage corroborated by presence of centrilobular necrosis, prominent neutrophilic infiltration and massive hemorrhage with fibrous expansion of portal tracts (Fig. 3C). Masson’s trichrome stained sections displayed deposition of collagen fibers with marked fibrous septa in day-14 NDEA treated rat, clearly showing the fibrogenesis (Fig. 3I). Similarly, Picrosirius red staining revealed the extent of collagen fiber deposition in both fluorescent microscopy and bright field microscopy in all the NDEA treated groups (Fig. 4). The NDEA intoxication showed brilliantly analyzed wide expanse of collagen fibers in red coloration under fluorescence microscope in day-7 specimens (Fig. 4H) however, the extent was much greater in the day-14 NDEA intoxicated animals (Fig. 4I) clearly indicating fibrosis. Treatment with PGJ showed rectification of the histological disruptions in a time and dose-dependent manner. A striking recuperation of the hepatic damage was apparent in PGJ supplemented groups which is evident by the scant observation of the red colored collagen in day-14 specimens (Fig. 4L), proposing antifibrotic potential of juice.

**Figure 5:**
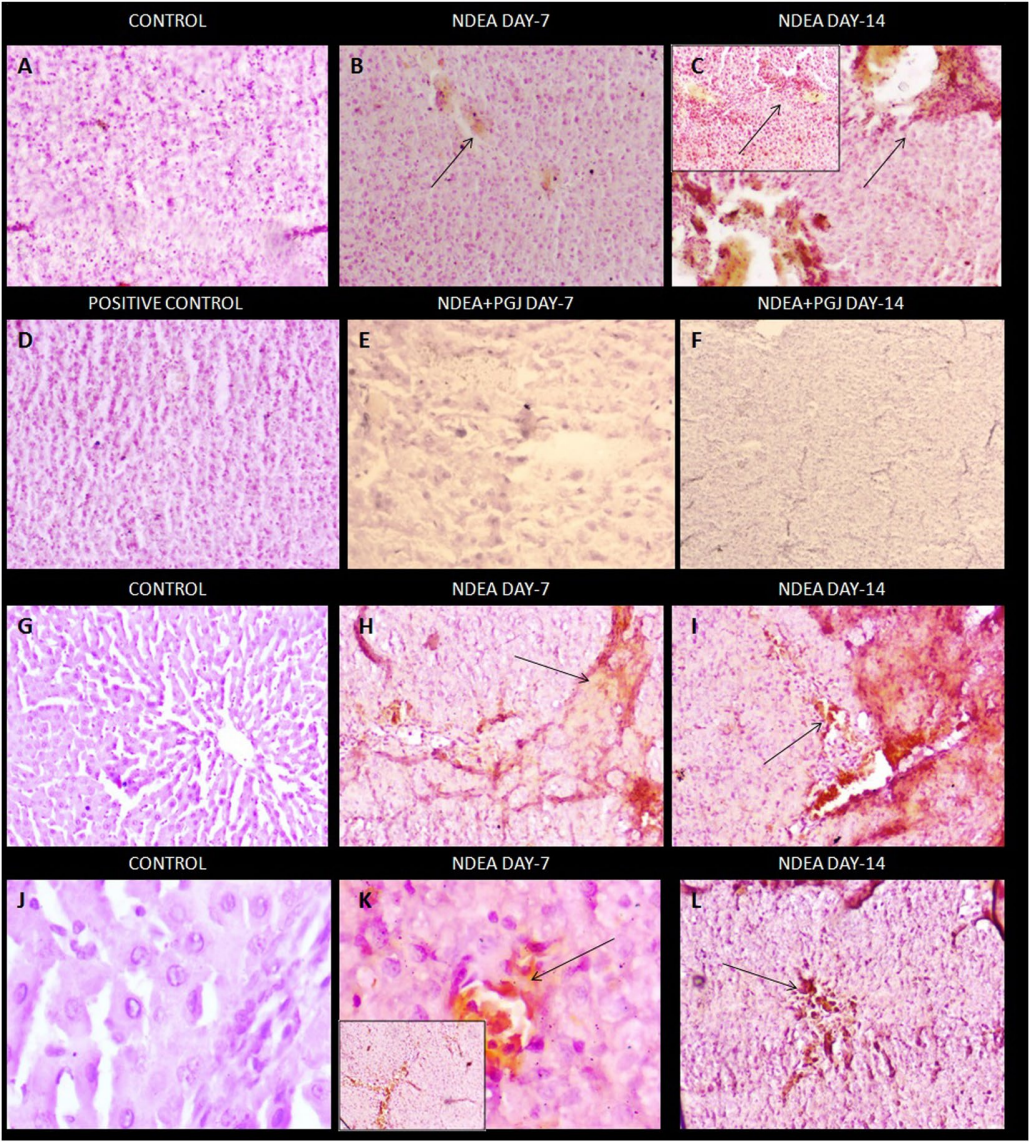
Immunohistochemical staining of COX-2. (**A**) Rat liver sections showing normal liver architecture with no staining of COX-2 (20X) (**B**). NDEA Day-7, COX-2 positive cells exhibiting damage (20X) (**C**) NDEA Day-14, Focal COX-2 staining of dark brown colour around excessively damaged area (20X). (Inset)- Positive staining of COX-2 in fibrotic region(20X) (**D**). Normal liver sections exhibiting typical lobular architecture and absence of COX-2positive staining (20X). (**E**). Immensely reduced binding of COX-2 antibody in rats treated with NDEA and PGJ in 7 days (40X) (**F**). NDEA + PGJ Day-14 liver specimens showing very scarce immunohistochemical staining (10X). Immunohistochemical staining of a-smooth muscle actin (a-SMA). (**G**) & (**J**). Normal liver showing a-SMA negative normal hepatocytes (20X & 40X). (**H**) & (**K**). NDEA Day-7 Myofibroblast-like cells in liver parenchyma representing activated hepatic stellate cells (20X & 40X). (**I**) & (**L**). NDEA Day-14 Activated stellate cells showing intense focal staining of a-SMA in fibrotic region (20X).

The localization of COX2, an inflammatory indicator and effecter enzyme of NFκB, was investigated by immunohistochemical staining of liver sections. In NDEA treated group, COX2 levels were elevated in the cytoplasm of most of the hepatocytes (Fig. 5B,C). In contrast control group lacked positive immune binding of COX-2 in all hepatocytes (Fig. 5D). PGJ supplemented liver sections reversed the effects of NDEA thereby decreasing the number of COX2 positive cells (Fig. 5E,F). The immunohistochemical staining of liver sections was carried out for the localization of α-SMA, a marker for activated hepatic stellate cells. Localization of α-SMA positive cells was seen around the periportal fibrotic band areas and also in the regions of connective tissue septa of NDEA treated rats (Fig. 5H,I,K & L). There were only insignificant number of α-SMA positive cells in the control liver sections (Fig. 5G,J).

**Figure 6:**
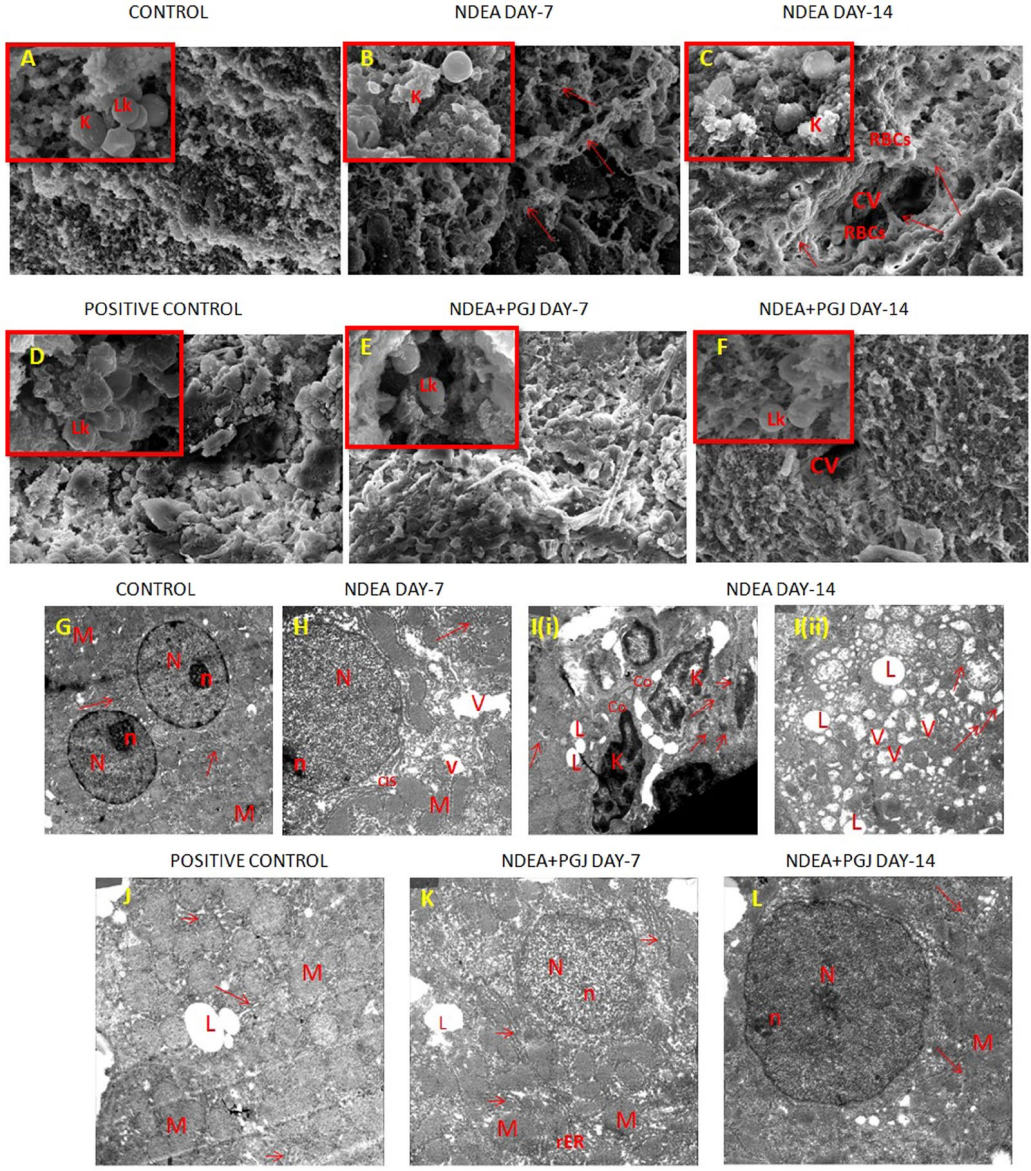
Scanning electron micrographs (SEM) of rat liver. **(A)** Saline control rat liver showing intact liver architecture without collagen fibres (1000X Scale-10 µm). (Inset)-Perfectly shaped kupffer cells and leukocytes (7000X Scale-2 µm). **(B)** NDEA Day-7 hepatic ultrastructure exhibiting collagen fiber formation depicted with arrows (1000X Scale-10 µm). (Inset)-Activated kupffer cells (7000X Scale-2 µM); **(C)** NDEA Day-14 treated section exhibiting hyperplasia and fibrosis around central vein. Collagen fibres are visibly thicker (1000X Scale-10 µm). (Inset)- Activated kupffer cells present (7000X Scale-2 µm). **(D)** PGJ control rat liver showing smooth cellular architecture without collagen (1000X Scale-10 µm). (Inset)-Smooth and unaltered leukocytes were also visible (7000X Scale-2 µm). **(E)** NDEA + PGJ Day-7 liver specimens exhibit reduced amount of collagen fibres (1000X Scale-10 µm). (Inset)- Distortions are absent in the leukocytes (7000X Scale-2 µm). **(F)** NDEA + PGJ Day-14 liver sections demonstrate profound central vein with normal architecture with no collagen fibers around it (1000X Scale-10 µm). (Inset) Leukocytes and cell types were also present in their typical shape (7000X Scale-2 µm). Ultrastructural changes in the rat liver observed under transmission electron microscope (TEM). **(G)** Saline control: Proper shaped nuclei with profoundly visible nucleolus and mitochondria. Arrows directing towards rough endoplasmic reticulum having short length (rER) (2000X Scale-2 µm). **(H)** NDEA Day-7 intoxicated rat liver showing distorted cell organelles: M = mitochondria with cristae and V = vacuoles observed more in number. Arrows indicate abnormally long rER (3000X Scale-500 nm). **(I)** NDEA Day-14 treated rat liver specimens showing **[i]** presence of numerous lipid droplets (L) and lysosomes (arrows), Collagen (Co) fibrils present in extra cellular matrix beside activated kupffer cells (K) (3000X Scale-500 nm) and **[ii]** depicting several vacuoles, lipid droplets, numerous condensed mitochondria, long and thick rER (3000X Scale-500 nm); **(J)** PGJ supplemented control liver sections exhibit typical cellular architecture with normal distribution of vacuoles, lipid droplets and mitochondria (3000X Scale-500 nm). **(K)** NDEA + PGJ Day-7 specimen showing slightly distorted nucleus, lesser number of lipid droplets (L) and vacuoles. Arrows showing shorter cisternae of rER and less condensed mitochondria indicating recovery phase (3000X Scale-500 nm). **(L)** NDEA + PGJ Day-14 liver sections demonstrate distinct nucleus and nucleolus with evidently less condensed mitochondria. Arrows showing normal shaped rER (3000X Scale-500 nm).

A three dimension architectural observation of the rat liver extracellular matrix from the control group showed some uneven, rough and discrete pebble like surfaces with no collagen fibers (Fig. 6A). The presence of normal kupffer cells and leukocytes were also detected in samples belonging to control groups (Fig. 6A-Inset). However, the NDEA treated day-7 and −14 samples exhibited various degrees of deleterious changes in liver architecture. Thin and thick both type of collagen fibers were visible in these sections and the surface appeared meshy with loss of integrity as compared to the control groups (Fig. 6B,C). Similarly, the obvious changes such as swelling, distortion and vesicles formation were evident in kupffer cells due to their activation by NDEA treatment (Fig. 6B-Inset & 6C-Inset). Further, our transmission electron microscopy results revealed normal hepatocyte nuclei, cytoplasm and short length rough endoplasmic reticulum (RER) in control groups (Fig. 6G). The hepatocytes in NDEA-treated animals showed multiple and distorted nuclei, irregular cytoplasm and increased number of mitochondria and vacuoles [Fig. 6H,I(i)]. The cytoplasm was characterized by enriched lipid droplets, increased lysosomes and condensed RER in NDEA intoxicated rats [Fig. 6I(ii)]. The effect of PGJ was evident by restoration of the overall liver architecture in the animals with lesser fibrous content, refurbished cell types, cytoplasm and cytoplasmic organelles in two weeks of treatment (Figs. 6K,L).

## Discussion

The utilization of phytoextract and nutraceuticals according to their appropriate recommended dosage has proven their health benefits and therapeutic potential. Here the chemo-preventive effect of pomegranate juice (PGJ) against the chemically-induced rat liver fibrosis has been explored. Pomegranate is rich in polyphenols and reported to have high antioxidant capacity thus has wide areas of possibilities for therapeutic usage^42^. Our study here explores the antifibrotic effect of PGJ in curbing the incidence of hepatic fibrosis induced by NDEA in Wistar rats. The liver fibrosis induced by NDEA, an environmental and dietary hepatocarcinogen, can be included as one of the best characterized experimental models of liver fibrosis helping in the establishment of various antifibrotic agents^29,66,67^. Published reports indicate that three weeks administration of Nitrosodimethylamine (NDMA/DMN) can also induce hepatic fibrosis in rodents^58,67^. However, NDEA may have an advantage over NDMA as the later requires more time and efforts.

The total phenolic content of PGJ used in this study manifested almost the same range ~1634 ± 34 mg/L as previously reported by Gil^68^; the DPPH radical scavenging (~55%) and FRAP assay (~1202 µM/L Fe^2+^) values also showed similarity with the levels already published on pomegranate juices from Turkey^69^. Previous studies have exhibited the action of pomegranate constituents in combating the chemically-induced tumors in skin, breast, lung and colon^70–75^. This study, thus indicate potential antioxidant capacity of PGJ that may be utilized as a cause of curbing the NDEA-induced hepatic fibrosis in rats.

Liver is an active participant involved in the metabolism of xenobiotics and drugs. Hepatocytes specifically play a major role in the detoxification of xenobiotics and drugs generating reactive oxygen species (ROS) and consequently causing oxidative stress. These redox disturbances have a wide impact on proteins, lipids and nucleic acids^50^. Extensive literature is available to document the imbalance between antioxidant defense system and generation of reactive oxygen species finally leading to oxidative stress. Therefore, in the present study investigations were made on the antioxidant status of the experimental animals exposed to NDEA. Superoxide dismutase (SOD) is an enzyme that incurs prominent defensive mechanism against superoxide radicals. NDEA significantly declines SOD within 14 days of its treatment. This decline in SOD activity is due to the oxidation of cysteine residues from the enzyme complex by the NDEA that ultimately leads to altered conformation of the enzyme and generation of superoxide radicals^76^. This is in accordance with the results related to declining SOD activity due to negative influences of oxidative stress^50^. The MDA levels were significantly increased in the liver of NDEA group further indicating oxidative stress. Lipid peroxidation of membrane fatty acids and a drastic increase in TBARS level culminate in the formation of MDA^50,77^. Our data reveals that pomegranate juice reversed the NDEA induced lipid peroxidation which significantly supports various other studies exhibiting the hepatoprotective effects of pomegranate in combating hepatic injury^3,78,79^.

The Ca^2+^, Mg^2+^ & Na^+^/K^+^ ATPases have been found to reach higher levels in liver fibrosis induced by NDEA^50,80^. These membranous ATPases were repudiated by pomegranate action which has never been reported before. Further, in the light of the observed levels of serum albumin which were found to be decreased, it is tempting to infer that NDEA induced liver fibrosis causes hypoalbuminaemia and pomegranate juice is a prominent player in restoring the levels back to normal. Our results are supported by relevant researches and are in concordance with quite a few studies which have also shown hypoalbuminaemia in cases of liver fibrosis as well as hepatocellular carcinoma^81–83^. The detoxifying and antioxidant enzymes such as catalase, GST, NQO1 provide protection against oxidative damage. Phase-I cytochrome (CYP) P450 enzymes yield electrophilic reactive products, CYP1A2 and CYP2E1 isozymes, that initiate the metabolism of NDEA leading to the generation of highly reactive electrophiles^84^. This oxidative electrophilic stress modifies the kelch-like erythroid cap ‘n’ collar homologue associated protein 1 (keap1) to which Nrf2 generally binds. This results in the dissociation of Nrf2 from keap1 and translocation of Nrf2 to the nucleus to bind to the antioxidant response element (ARE) in the promoter region of target genes^85^. This ultimately may leads to the synthesis of antioxidant enzymes. The dissociation of Nrf2-Keap1 complex occurs when exposed to internal oxidative stress or external stimuli such as UV radiations, xenobiotics, carcinogens etc^86^. Thus NDEA may also induce Nrf2 translocation to the nucleus. However, the role of NDEA is also to upregulate NFкB in cells^87^ which further paves the way for the cross-talk between NFкB and Nrf2. In cases when NFкB and Nrf2 are simultaneously activated, NFкB acts antagonistically on Nrf2^88^. Ultimate downregulation of the protein levels of Nrf2 and HO-1 have been observed by the administration of NDEA in mice^87^. It has been reported about naturally occurring dietary polyphenols that they can upregulate antioxidant genes by activating Nrf-2, regulate signaling pathways *via* NFкB and MAP kinase, beyond their free radical scavenging activity^89^. Interaction between the two proteins Nrf2 and NFκB is an established phenomena in inflammation and carcinogenesis^90^. As far as for induction of NFκB it is imperative to mention that the proinflammatory biomarkers like COX2, iNOS, TNFα all of which belong to the effecter genes category controlled by the NFκB pathway have been documented to show more pronounced induction in Nrf2 deficient mice in comparison to the wild type mice. This evidently concludes that NFκB mediated proinflammatory reactions are accelerated by the eviction of Nrf2^91^. In light of which our results of Immunohistochemistry of COX2 evidently support this inference. The group NDEA + PGJ has a higher level of Nrf2 and lower levels of COX2 antibody binding. This is also reaffirmed as COX2 is rate limiting enzyme for the synthesis of 15d-PGJ_2_ (prostaglandin) and when COX2 is inhibited then Nrf2 transactivation is repressed^92^.

The observation that PGJ upregulates these levels are an indicative of the important role played by polyphenolic compounds in quenching the free radicals generated by NDEA metabolism. Thus, supplementation of PGJ has an antifibrotic effect through Nrf2 upregulation and modulating the cross-talk between transcription factors Nrf2 and NFκB^88^. Our findings underline the notion that Nrf2 is one of the major cellular defense lines against oxidative stress as it mediates antioxidant response by promoting NADPH production through pentose phosphate pathway^93^. Oxidative stress leads to triggering of Nrf2 that upregulates the rate limiting enzyme G6PD in the pentose phosphate pathway^94^. Levels of G6PD under conditions of oxidative stress are increased interestingly^95–98^. Our results demonstrate increased levels of G6PD activity in the NDEA intoxicated fibrotic rats. The increase in G6PD activity during stress conditions is a defensive act for fulfilling the inhibition of NADPH done by ROS^58,95^.

**Figure 7:**
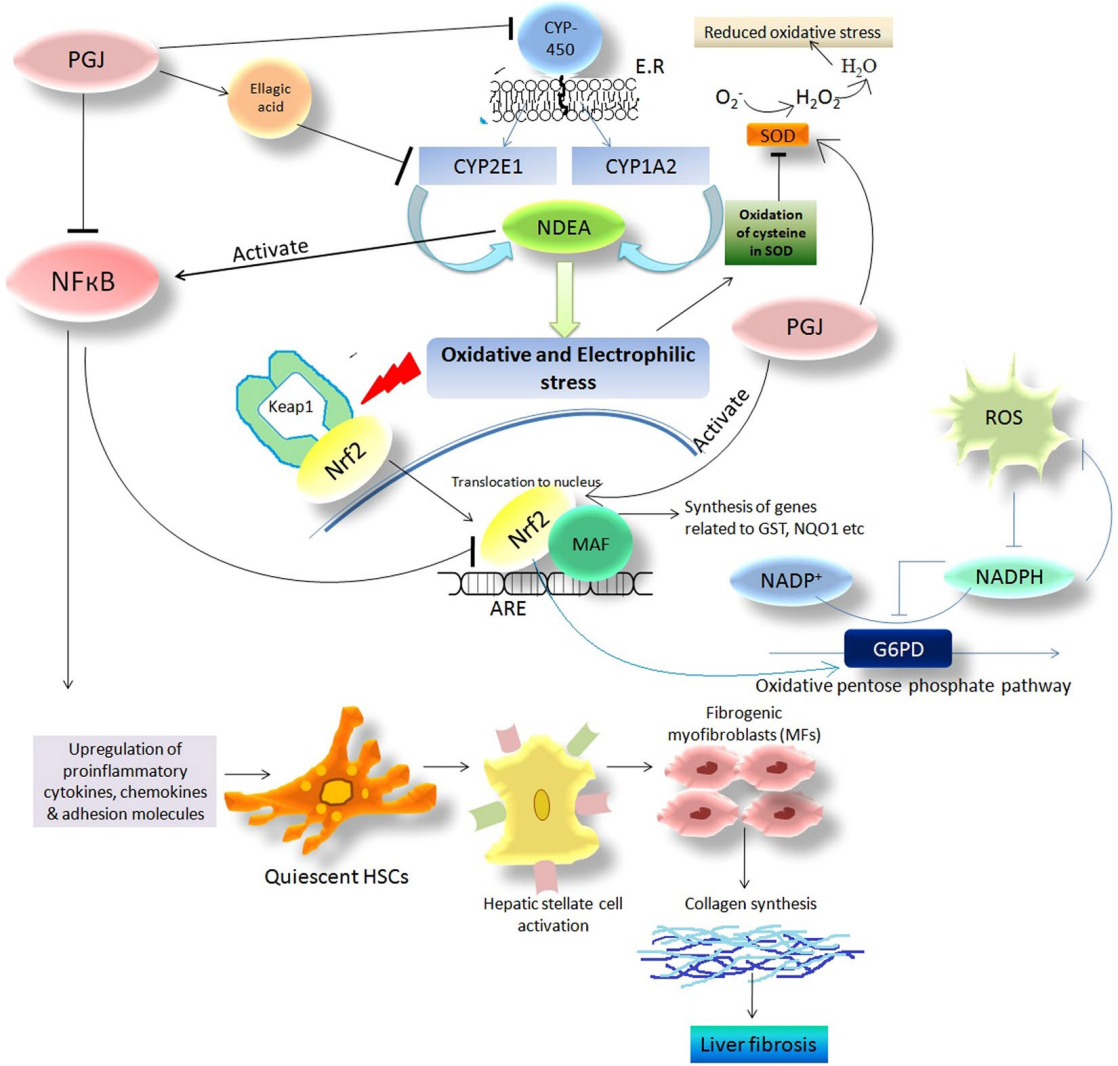
Proposed mechanism of amelioration by PGJ against NDEA induced liver fibrosis.

Our data also demonstrate a decline in the levels of cytosolic Nrf2 in NDEA treated fibrotic animals (day-14), which is in agreement with other published reports^40,87^. In animals supplemented with PGJ, Nrf-2 levels refurbished significantly within two weeks indicating protective effect of PGJ which is probably mediated *via* declining oxidative stress in rats. Nevertheless pomegranate’s retaliation to this damage of catalase and glutathione levels is indicative of utilization of its antioxidant activity in the mechanism against hepatic fibrosis. An interesting report by Ahn^99^ also demonstrate that ellagic acid, a component of PGJ leads to a decline in the levels of both the total hepatic CYP450 and CYP2E1 clearly indicate a correlation of PGJ action on the deactivation of NDEA-induced oxidative stress. The interaction of Nrf2 with NFкB significantly predicts that Nrf2 may act as major mediator in inflammatory pathways^100^ (Fig. 7).

Our results based on H-E, Picrosirius, Masson’s trichrome stainings further documents gross alterations in the anatomy of fibrotic livers. These histopathological examinations converge to give out the result as NDEA treated rat livers have an excess of hepatocyte necrosis, excessive collagen, dilated sinusoidal spaces and neutrophilic infiltration at the site of inflammation. Hence liver inflammation is prominent in the NDEA treated group. Time-dependent severity of liver inflammation followed by fibrosis in our study is well represented by the increased sera AST, ALT, ALP, γGT and bilirubin levels caused by the administration of NDEA^50,101,102^. These liver function biomarkers have cytoplasmic origin and after cellular damage by NDEA get released into the circulation^54,84,102^. However, the PGJ significantly restored the levels of these liver indices which were further confirmed by reduction in the extent of histopathological damage.

In our study the scanning electron microscopy observations and histopathological examinations by Masson’s trichrome and picro-sirius staining showed the presence of excess collagen in the liver sections intoxicated by NDEA; which is a clear indication of liver fibrosis and inflammation. Cytokines, growth factors and other chemical messengers are secreted by inflammatory immune cells which in turn activate hepatic stellate cells that lead to the synthesis of collagen^31^. Our observations on the scanning electron microscopy of liver sections coincide with the results of the transmission electron microscopy images. The ultrastructural changes of remarkable nature were found in mitochondria and endoplasmic reticulum of the hepatocytes. The hepatocytes of fibrotic liver consisted of denser endoplasmic reticulum and swollen mitochondria with disrupted structure of cristae. This loss of normal cristae structure and dense mitochondria is indicative of derogative mitochondrial function^103–105^. Diversely sized and dilated rough endoplasmic reticulum as observed in day-14 NDEA treated liver specimens represents severely damaged hepatocytes^106^. These ultrastructural changes in mitochondria and endoplasmic reticulum can be related with liver fibrosis as they both produce ROS (reactive oxygen species) in the liver via cytochrome P450 enzymes^107,108^. Major cells hepatocytes are extremely active in the metabolism of xenobiotics and drugs^41^. Hepatocyte cell lines the HepG2 cells have been known to exhibit the activities of Nrf2 and NFκB^109,110^. During the process of liver fibrosis hepatic stellate cells get activated which results in trans-differentiation into myofibroblasts phenotype. This leads to an abnormal extracellular matrix synthesis and expression of α-SMA gene^50^. Our experiments in this study on immunohistochemistry of α-SMA in NDEA treated rats evidently show that hepatic stellate cells are the key cell type involved in NDEA-induced liver fibrosis.

These results provide evident insights into the ultra-structural changes of the cells undergoing damage in hepatic fibrosis and furthermore undergoing recovery by pomegranate supplementation. Very few studies are there on the SEM and TEM micrographs of the liver tissue undergoing chemically induced hepatic fibrosis^63,106^. Our results, thus strongly support these studies as they also exhibit similar amount of damage incurred during liver fibrosis.

In conclusion this communication exhibits the action of pomegranate in curbing the oxidative stresses by abatement of superoxide dismutase, glutathione and catalase levels. The abrogation of membrane ATPases, liver function indices and lipid peroxidation levels all indicate the recuperative potential of pomegranate against NDEA induced liver fibrosis. Furthermore the histopathological studies utilizing the staining by H&E, M&T, picro-sirius in bright field microscopy and fluorescence microscopy; evidently indicate the regression of liver fibrosis by PGJ. Electron microscopy and electrophoretic techniques utilized in this study all decisively direct to the probability of involvement of Nrf-2 in the pomegranate mechanism of action. Hence we propose a possible mechanism of action of pomegranate against NDEA induced liver fibrosis which mediates *via* upregulation of Nrf-2 and down-regulation of NFκB.
